# Lipid Biomarkers and Cardiometabolic Diseases: Critical Knowledge Gaps and Future Research Directions

**DOI:** 10.3390/metabo15020108

**Published:** 2025-02-07

**Authors:** Hyun Suk Yang

**Affiliations:** Department of Cardiovascular Medicine, Research Institute of Medical Science, Konkuk University School of Medicine, Seoul 05029, Republic of Korea; yang.hyun@kuh.ac.kr; Tel.: +82-2-2030-7519

The past decade has witnessed transformative changes in our understanding of various lipid or lipid-related biomarkers (Table 1) and their relationships with cardiometabolic diseases. This evolution has been driven by technological advances in lipidomics and a deeper appreciation of the complex interplay between various metabolic pathways. Several technological advances have been made in this regard. High-resolution mass spectrometry techniques have enabled enhanced lipid separation, identification, and comprehensive profiling [1,2]. Complementarily, nuclear magnetic resonance spectroscopy facilitates the detailed characterization of lipoprotein particle distributions, including the quantitative analysis of particle numbers and sizes, thereby enabling robust subclass determination [3,4]. The detection of novel lipid mediators such as ceramides, oxidized phospholipids, and fatty acid derivatives has been reported to be a key mediator of metabolic dysfunction [5,6,7]. The integration of lipidomics with other omics data (proteomics and metabolomics) has expanded the lipid research area [3,8,9], with the integration of machine learning approaches for biomarker pattern recognition [10,11]. Clinically, paradigm shifts have occurred in disease classification to embrace a more nuanced, metabolism-based definition. For example, the recent transition from non-alcoholic fatty liver disease (NAFLD) to metabolic dysfunction-associated steatotic liver disease (MASLD) reflects metabolic pathophysiology under these conditions [12,13]. Similarly, the concept of cardio–kidney–metabolic (CKM) syndrome has emerged, which indicates a connection between cardiovascular, renal, and metabolic diseases [14,15].

## 1. Special Issue Contribution

This Special Issue on “Lipid Biomarkers and Cardiometabolic Diseases” features five articles covering advances in the role of various lipid biomarkers, from cardiometabolic disease risk evaluation to interventional guidance.

Abu-Farha et al. (August 2023) (Contribution 1) provides novel mechanistic insights into how the ANGPTL8 R59W variant influences metabolic pathways and inflammation in Arab populations. A targeted analysis of 408 plasma metabolites revealed significant alterations in key metabolic mediators among the variant carriers, including the dysregulation of acylcarnitines, phosphatidylcholines, cholesteryl esters, and α-aminoadipic acid. Particularly noteworthy were the increased acylcarnitine and decreased phosphatidylcholine concentrations in the variant carriers, suggesting perturbations in the metabolic pathways and inflammation. These metabolic signatures expand our understanding of ANGPTL8′s role beyond simple lipid regulation and demonstrate its broad influence on inflammatory cascades that may contribute to cardiometabolic risk.

A study by Yang et al. (November 2023) (Contribution 2) brought clinical importance to the sex-specific approach to HDL cholesterol analysis. Korean adults (1.7 million) with atherosclerotic cardiovascular disease showed a slightly U-shaped correlation between HDL cholesterol levels and the 10-year mortality rate, with an earlier inflection in males (nadir: 50–59 mg/dL) than in females (nadir: 70–79 mg/dL). The extremely high HDL cholesterol group (>90 mg/dL) had an increased risk of 10-year all-cause mortality by 35.9% in males and 9.5% in females compared with the reference group (males: 40–90 mg/dL, females: 50–90 mg/dL). These findings challenge the conventional HDL cholesterol paradigm and underscore the importance of sex-specific reference ranges in cardiovascular risk stratification.

Choi et al. (March 2024) (Contribution 3) reported incremental value of Apolipoprotein B (ApoB) testing as a complement to traditional lipid panels for cardiovascular disease risk assessment in 9604 Korean adults. Using the NCEP ATP III criteria, traditional lipid panels identified a 27.4% prevalence of dyslipidemia. Based on an ApoB cutoff of ≥100 mg/dL, up to 17.5% of the participants exhibited isolated high ApoB levels in the absence of traditional lipid test anomalies. This suggests that incorporating ApoB testing could enhance cardiovascular disease risk stratification in the Korean population.

A comprehensive review by Kounatidis et al. (February 2024) (Contribution 4) further explored the pivotal role of ApoB100 in the pathogenesis of atherosclerosis. The authors detail ApoB100′s structure, metabolism, and function as the primary protein component of atherogenic lipoproteins, including VLDL, IDL, LDL, and Lp(a). This elucidates the molecular mechanisms by which ApoB100 contributes to atherosclerotic plaque formation, particularly focusing on its interactions with proteoglycans in the subendothelium and its involvement in inflammatory cascades through NLRP3 inflammasome activation. Additionally, the review evaluated ApoB100′s emerging value as a superior predictor of cardiovascular risk compared to traditional LDL cholesterol measurements, especially in patients with residual cardiovascular risk despite standard lipid-lowering therapy.

Another review by Kounatidis et al. (July 2024) (Contribution 5) provides a comprehensive analysis of the non-lipid effects of various lipid-modifying therapies. The authors systematically examined traditional agents such as statins and ezetimibe, along with newer therapeutics such as PCSK9, bempedoic acid, and ANGPTL3 inhibitors. This study details how these interventions influence multiple physiological pathways including inflammation, vascular function, glucose metabolism, and cancer development. Particular attention has been paid to emerging evidence supporting the therapeutic potential of HDL cholesterol enhancement strategies, including CETP inhibitors and recombinant HDL particles, and the pleiotropic benefits of agents used in hypertriglyceridemia management, such as fibrates, omega-3 fatty acids, and ApoCIII inhibitors. The review emphasizes that understanding these pleiotropic effects is crucial for optimizing therapeutic strategies and potentially expanding the clinical applications of these agents beyond cardiovascular disease management.

## 2. Critical Knowledge Gaps

Several critical knowledge gaps in lipidomics require further investigation. While traditional lipid biomarker analysis has focused predominantly on measuring the concentrations of LDL cholesterol, HDL cholesterol, and triglycerides, this conventional approach fails to capture the intricate dynamics and functionality of lipoprotein particles across diverse metabolic states, potentially compromising the accuracy of risk stratification. Novel lipid biomarkers (including ApoB [16], small dense LDL particles [17], lipoprotein(a) [18], and omega-3 fatty acids [7]) or regulatory molecules serving as biomarkers (such as ANGPTL [19], CETP [20], PCSK9 [21], and non-coding RNAs [22,23]) face their own challenges. These include a lack of standardized measurement methods, limited validation across large, diverse populations, and an incomplete understanding of their relationships or casualties with cardiometabolic diseases. Additionally, uncertainty remains regarding how to integrate multiple biomarkers cost-effectively in clinical practice.

The interplay between multiple metabolic conditions presents a complex challenge in lipidomic research [24]. A significant knowledge gap exists in the understanding of the mechanistic relationship between MASLD and cardiovascular manifestations [12,13,25]. Moreover, CKM syndrome remains not fully characterized worldwide, with substantial gaps in epidemiological data [15]. Despite mounting evidence of sex-based differences in lipid metabolism and cardiovascular risk profiles, the widely used LDL-cholesterol equations have a sex-agnostic approach [26,27]. Additionally, the field lacks robustly validated sex- or ethnic-specific lipid threshold values [28,29], potentially compromising the accuracy of risk assessments across diverse populations.

## 3. Future Research Directions

The rapidly evolving field of lipid biomarkers and cardiometabolic diseases has provided numerous compelling research opportunities. First, the integration of multiomics approaches offer promising avenues for investigation [3,8,9]. This includes the synthesis of lipidomics with genomics and proteomics, exploration of lipid–microbiome interactions, and development of comprehensive multi-parametric models. Second, population-specific research demands attention, particularly in large-scale validation studies examining sex-, ethnic-, and age-related variations in lipid biomarkers. Third, therapeutic target identification remains crucial, encompassing biomarker-guided therapeutic approaches, evidence-based dietary recommendations, and applications in personalized precision medicine. Fourth, clinical translation warrants investigation of the shared pathways between inflammation and various cardiometabolic conditions [30].

## 4. Call for Future Contributions

We extend this enthusiastic invitation to researchers to address these critical knowledge gaps and contribute to emerging areas of investigation. The forthcoming second edition of our Special Issue focuses on these themes and welcomes innovative studies to advance our understanding of lipid biomarkers in cardiometabolic diseases. We particularly welcome original research articles investigating novel biomarker applications and validations as well as methodological papers introducing innovative analytical approaches and technical advances. Additionally, we encourage the submission of clinical studies evaluating biomarker utility in diverse patient populations, position papers proposing novel conceptual frameworks and paradigm shifts, and comprehensive reviews synthesizing the current knowledge and future directions in lipid biomarker research.

The field of lipid biomarkers continues to offer abundant opportunities for transformative research that can significantly impact patient care. We anticipate your valuable contributions to our upcoming Special Issues.

## Figures and Tables

**Table 1:** Classification of lipid or lipid-related biomarkers for cardiometabolic diseases.

Class	Sub-Class	Examples
Sterols	Cholesterol	
	Lipoproteins	HDL, LDL, VLDL, IDL, Lp(a); Subtypes, small dense LDL particles
	Apolipoproteins	ApoB, ApoA-I
Glycerolipids	Triglycerides	
	Diglycerides	
	Monoglycerides	
Phospholipids	Glycerophospholipids	
	Lysophospholipids	
Sphingolipids	Ceramides	Cer(d18:1/16:0), Cer(d18:1/18:0), Cer(d18:1/24:1)
	Sphingomyelins	SM(d18:1/16:0), SM(d18:1/18:0), SM(d18:1/24:1)
	Glycosphingolipids	Glucosylceramides, Lactosylceramides
Fatty acids	Saturated fatty acids	Palmitic acid, Stearic acid
	Unsaturated fatty acids	Omega-3, Omega-6 fatty acids
Eicosanoids	Prostaglandins	
	Leukotrienes	
	Thromboxanes	
Oxidized Lipids	Oxidized phospholipids	
	Oxidized cholesterol	
	Oxidized fatty acids	
Lipid Metabolism Regulators	ANGPTL	ANGPTL3, ANGPTL4, ANGPTL8
	Adipokines	Adiponectin
	CETP	
	LPL	
	PCSK9	
Non-coding RNAs	miRNAs	miR-33, miR-122, miR-27
	IncRNAs	CHROME, LeXis
Lipid Transport Protein	ABCA1	
	ABCG5/G8	
	NPC1L1	
	SRB1	
Inflammatory Markers Related to Lipid Metabolism	Lipoprotein-associated phospholipase A2	
	Myeloperoxidase	
	Serum Amyloid A	

Abbreviations: HDL: high-density lipoprotein; LDL: low-density lipoprotein; VLDL: very-low-density lipoprotein; IDL: intermediate-density lipoprotein; Lp(a): lipoprotein(a); Apo: apolipoprotein; Cer: ceramide; SM: sphingomyelin; ANGPTL: angiopoietin-like protein; CETP: cholesteryl ester transfer protein; LPL: lipoprotein lipase; PCSK9: proprotein convertase subtilisin/kexin type 9; miRNA: microRNA; lncRNA: long non-coding RNA; CHROME: cholesterol homeostasis regulator of microRNA expression; LeXis: liver-expressed LXR-induced sequence; ABCA1: ATP-binding cassette transporter A1; ABCG5/G8: ATP-binding cassette transporters G5/G8; NPC1L1: Niemann–Pick C1-like 1; SRB1: scavenger receptor class B type 1.
